# On the origin and diversification of Podolian cattle breeds: testing scenarios of European colonization using genome-wide SNP data

**DOI:** 10.1186/s12711-021-00639-w

**Published:** 2021-06-02

**Authors:** Gabriele Senczuk, Salvatore Mastrangelo, Paolo Ajmone-Marsan, Zsolt Becskei, Paolo Colangelo, Licia Colli, Luca Ferretti, Taki Karsli, Hovirag Lancioni, Emiliano Lasagna, Donata Marletta, Christian Persichilli, Baldassare Portolano, Francesca M. Sarti, Elena Ciani, Fabio Pilla

**Affiliations:** 1 Department of Agricultural, Environmental and Food Sciences, University of Molise, 86100 Campobasso, Italy; 2 Department of Agricultural, Food and Forest Sciences, University of Palermo, 90128 Palermo, Italy; 3 Department of Animal Science Food and Nutrition, DIANA, Nutrigenomics and Proteomics Research Centre, PRONUTRIGEN, Biodiversity and Ancient DNA Research Centre, BioDNA, Università Cattolica del Sacro Cuore, Piacenza, Italy; 4 Department of Animal Breeding and Genetics, Faculty of Veterinary Medicine, Bulevar Oslobodjenja street 18, 11000 Belgrade, Serbia; 5 National Council of Research (CNR), Research Institute On Terrestrial Ecosystems (IRET), Via Salaria km 29.300, Montelibretti, 00015 Rome, Italy; 6 Department of Biology and Biotechnology, University of Pavia, Italy Pavia,; 7 Department of Animal Science, Faculty of Agriculture, Akdeniz University, Antalya, Turkey; 8 Department of Chemistry, Biology and Biotechnology, University of Perugia, via Elce di sotto, 06123 Perugia, Italy; 9 Department of Agricultural, Food and Environmental Sciences, University of Perugia, 06121 Perugia, Italy; 10 Department of Agriculture, Food and Environment, University of Catania, 95125 Catania, Italy; 11 Department of Bioscience, Biotechnology and Biopharmaceuticals, University of Bari, 70124 Bari, Italy

## Abstract

**Background:**

During the Neolithic expansion, cattle accompanied humans and spread from their domestication centres to colonize the ancient world. In addition, European cattle occasionally intermingled with both indicine cattle and local aurochs resulting in an exclusive pattern of genetic diversity. Among the most ancient European cattle are breeds that belong to the so-called Podolian trunk, the history of which is still not well established. Here, we used genome-wide single nucleotide polymorphism (SNP) data on 806 individuals belonging to 36 breeds to reconstruct the origin and diversification of Podolian cattle and to provide a reliable scenario of the European colonization, through an approximate Bayesian computation random forest (ABC-RF) approach.

**Results:**

Our results indicate that European Podolian cattle display higher values of genetic diversity indices than both African taurine and Asian indicine breeds. Clustering analyses show that Podolian breeds share close genomic relationships, which suggests a likely common genetic ancestry. Among the simulated and tested scenarios of the colonization of Europe from taurine cattle, the greatest support was obtained for the model assuming at least two waves of diffusion. Time estimates are in line with an early migration from the domestication centre of non-Podolian taurine breeds followed by a secondary migration of Podolian breeds. The best fitting model also suggests that the Italian Podolian breeds are the result of admixture between different genomic pools.

**Conclusions:**

This comprehensive dataset that includes most of the autochthonous cattle breeds belonging to the so-called Podolian trunk allowed us not only to shed light onto the origin and diversification of this group of cattle, but also to gain new insights into the diffusion of European cattle. The most well-supported scenario of colonization points to two main waves of migrations: with one that occurred alongside with the Neolithic human expansion and gave rise to the non-Podolian taurine breeds, and a more recent one that favoured the diffusion of European Podolian. In this process, we highlight the importance of both the Mediterranean and Danube routes in promoting European cattle colonization. Moreover, we identified admixture as a driver of diversification in Italy, which could represent a melting pot for Podolian cattle.

**Supplementary Information:**

The online version contains supplementary material available at 10.1186/s12711-021-00639-w.

## Background

The tangled evolutionary history of cattle stems from two main wild aurochs subspecies, *Bos primigenius primigenius* and *Bos primigenius namadicus* which were widespread across Africa and Eurasia during Middle Pleistocene. They diverged approximately 250,000 years before present (YBP) and were independently domesticated giving rise to the current *Bos taurus taurus* (taurine) and *Bos taurus indicus* (zebuine) subspecies*,* respectively [1, 2]. Since domestication, which occurred about 10,000 YBP in the Fertile Crescent (*B.t. taurus*) and about 8000 YBP in the Indus Valley (*B.t. indicus*), cattle followed colonization routes in parallel with the Neolithic human expansions [3, 4]. Indeed, 4000 years later, traces of zebu introgression into the taurine genome were already present in cattle from a region spanning from Central Asia and Iran to the Caucasus and Mediterranean shores [5]. Such an important zebuine component in taurine genomes may have been favoured and maintained by the capacity of zebus to adapt to harsh and arid environments [6]. The westward human-mediated expansion of cattle also led to hybridization events between domesticated cattle and wild *Bos primigenius* present in both Europe and Africa [4]. Although this general picture is well established, studies using either mitochondrial genome or nuclear single nucleotide polymorphism (SNP) data revealed greater complexity. Indeed, based on the high level of genetic divergence of the African taurine cattle, it has been argued that a third independent domestication event in North-East Africa (about 8000–9000 YBP) may have occurred [7, 8], although recent studies based on genome-wide SNPs seem to refute this hypothesis [4, 9]. Therefore, the post-domestication evolutionary history of cattle is characterized by a number of ancient introgression events that were driven by both neutral and adaptive processes and more recent crosses ruled by human selection purposes, which on the whole gave rise to remarkably high levels of breed diversity in Europe. Concerning mitochondrial DNA evidence, the T clade is the most represented mitochondrial lineage in both modern and ancient Neolithic European cattle [10–12] and is believed to have been captured during the domestication event in the Fertile Crescent, but other rare lineages are also scattered throughout Europe. For example, the P lineage is mainly present in central and northern European aurochs, and in a small number of modern taurine breeds and ancient European domestic cattle and the Q lineage in several Italian, Egyptian and Neolithic European cattle [13], whereas the rare R lineage is found only in some of the Italian cattle breeds considered to belong to the so-called Podolian trunk and in a sample of wild aurochs from Morocco [5, 14–20]. On the one hand, the basal phylogenetic position occupied by these clades within the taurine radiation strongly supports Europe as an important centre of diversification of wild aurochs’ populations during the late Pleistocene, which later intermingled with domesticated cattle [8]. This intriguing pattern led some authors to suggest that Italy might have been the site of an additional domestication or at least introgression event involving local aurochs [21]. On the other hand, analysis of genomic data at the global scale has led to another picture, indicating three main clusters (*B. t. indicus*, European *B. t. taurus* and African *B. t. taurus*) with different levels of genetic admixture among them [4, 9, 22].

The European cattle gene pool is mainly of *B. t. taurus* origin. However, an indicine component was observed in diverse breeds, such as the Turkish Grey and some Italian breeds that belong to the so-called Podolian trunk (Chianina, Romagnola, Marchigiana, Maremmana and Podolica Italiana) and show evidence of mixed *B. t. taurus* and *B. t. indicus* ancestries [22–24].

Cattle breeds belonging to the so-called Podolian trunk, also known as grey steppe cattle (hereafter referred to as “Podolian”) are distributed across continental Europe and the Mediterranean basin, and represent a fundamental resource and income for traditional farming thanks to local products. In general, these breeds, compared with other European cattle breeds, still display many ancestral features such as long horns, sexual dimorphism, longevity, and are well adapted to the local climatic and environmental conditions. However, their history and origin are still not well established, although several hypotheses have been proposed to explain the origin of Podolian cattle. Analysis of mitochondrial DNA data suggests an Anatolian and South-Western Asian origin of Central Italian Podolian breeds, docked to Italy via the sea route, probably during the Etruscan period [20, 25]. Alternatively, Podolian cattle, that reached the Balkans and Central Europe in parallel with human expansion, returned South more recently, likely during the barbarian invasions. Notably, although indicine introgression into the Podolian genome has long been considered as ancestral, tracing back to the early Neolithic expansion, analysis of ancient DNA revealed that it occurred more recently, about 4200 YBP [5], which opens up new perspectives on modes and times of European cattle colonization.

To disentangle this complex history, we used genome-wide SNP data to specifically: (i) reconstruct the origin and diversification of the Podolian cattle breeds by comparing them with other European taurine, African taurine and Asian indicine cattle breeds, (ii) better understand the genomic relationships within Podolian cattle breeds, and (iii) provide the most plausible scenario of European colonization, through an approximate Bayesian computation random forest (ABC-RF) approach.

## Methods

### Samples

We used a set of 806 individuals belonging to 36 cattle breeds genotyped with the Illumina BovineSNP50 (Table 1) and (see Additional file 1: Table S1). The dataset included six zebuine breeds, seven African taurine breeds and 23 European taurine breeds. Since no unambiguous criterion is available to classify the Podolian breeds, in this paper, we use the term Podolian with a broad connotation including all the breeds which have been either traditionally acknowledged as of Podolian origin or display phenotypic traits that are shared among Podolian breeds (grey coat, fawn pigmentation of the calf at birth and large horns). Among these, we included the grey steppe breeds, mainly from continental Europe and the Balkans (Bulgarian Grey, Boskarin, Croatian Podolian, Hungarian Grey, Serbian Podolsko, Romanian Grey and Ukrainian Grey), Podolian breeds from Italy (Calvana, Chianina, Cinisara, Modicana, Marchigiana, Maremmana, Podolica Italiana and Romagnola), and other South-East Mediterranean breeds usually acknowledged as of Podolian origin (Turkish Grey and Katerini) or with phenotypic traits recalling those of most Podolian cattle (Guelmoise and Gascon).

**Table 1:** Genetic diversity indices for the analyzed cattle breeds

	Breed name	Code	N	Ho ± SD	He ± SD	MAF ± SD	Ne
1	Hariana	HAR	10	0.145 ± 0.217	0.138 ± 0.186	0.102 ± 0.151	21
2	Gabrali	GBI	10	0.216 ± 0.207	0.207 ± 0.179	0.147 ± 0.149	48
3	Gir	GIR	20	0.183 ± 0.190	0.184 ± 0.182	0.132 ± 0.149	90
4	Sahiwal	SAHW	17	0.186 ± 0.197	0.183 ± 0.183	0.131 ± 0.150	68
5	Lohani	LOH	10	0.190 ± 0.200	0.187 ± 0.181	0.133 ± 0.148	52
6	Ongole Grade	ONG	20	0.243 ± 0.190	0.237 ± 0.168	0.166 ± 0.143	52
7	Kuri	KUR	20	0.292 ± 0.197	0.286 ± 0.178	0.214 ± 0.159	105
8	N'Dama	NDAM	51	0.274 ± 0.188	0.275 ± 0.181	0.205 ± 0.160	181
9	Baoule	BAO	20	0.267 ± 0.211	0.254 ± 0.189	0.190 ± 0.163	90
10	Somba	SOM	20	0.262 ± 0.194	0.269 ± 0.185	0.201 ± 0.161	105
11	Lagune	LAG	20	0.229 ± 0.213	0.224 ± 0.197	0.168 ± 0.166	83
12	Oulmès Zaer	OUL	19	0.335 ± 0.191	0.318 ± 0.163	0.240 ± 0.152	55
13	Guelmoise	GUE	24	0.324 ± 0.166	0.338 ± 0.148	0.256 ± 0.145	138
14	Turkish Grey	TUG	24	0.348 ± 0.164	0.348 ± 0.142	0.263 ± 0.142	117
15	Katerini	KAT	19	0.319 ± 0.157	0.352 ± 0.143	0.263 ± 0.146	82
16	Bulgarian Grey	BLG	20	0.327 ± 0.203	0.301 ± 0.172	0.226 ± 0.156	44
17	Romanian Grey	ROG	4	0.351 ± 0.299	0.261 ± 0.192	0.198 ± 0.168	15
18	Serbian Podolsko	PDS	24	0.294 ± 0.213	0.274 ± 0.188	0.204 ± 0.164	33
19	Croatian Podolian	CRP	24	0.331 ± 0.193	0.312 ± 0.167	0.235 ± 0.154	48
20	Hungarian Grey	HUG	24	0.338 ± 0.181	0.329 ± 0.159	0.249 ± 0.150	77
21	Ukrainian Grey	UKG	48	0.346 ± 0.168	0.335 ± 0.154	0.193 ± 0.168	75
22	Boskarin	BOS	30	0.351 ± 0.162	0.347 ± 0.144	0.263 ± 0.143	108
23	Cinisara	CIN	30	0.349 ± 0.155	0.354 ± 0.140	0.270 ± 0.141	133
24	Modicana	MOD	30	0.338 ± 0.171	0.334 ± 0.154	0.253 ± 0.147	106
25	Podolica Italiana	POD	24	0.351 ± 0.157	0.356 ± 0.138	0.272 ± 0.140	122
26	Maremmana	MRM	25	0.338 ± 0.192	0.319 ± 0.165	0.242 ± 0.153	59
27	Marchigiana	MRC	22	0.347 ± 0.172	0.341 ± 0.149	0.258 ± 0.145	96
28	Chianina	CHN	23	0.334 ± 0.176	0.330 ± 0.155	0.249 ± 0.148	88
29	Calvana	CAL	24	0.314 ± 0.196	0.301 ± 0.173	0.226 ± 0.156	56
30	Romagnola	RMG	21	0.333 ± 0.181	0.324 ± 0.159	0.244 ± 0.149	72
31	Modenese	MDN	24	0.348 ± 0.172	0.340 ± 0.151	0.258 ± 0.146	76
32	Piedmontese	PMT	21	0.367 ± 0.166	0.356 ± 0.138	0.271 ± 0.140	112
33	Tyrolean Grey	TYR	24	0.350 ± 0.175	0.338 ± 0.152	0.256 ± 0.147	84
34	Gascon	GAS	20	0.346 ± 0.176	0.339 ± 0.152	0.258 ± 0.146	96
35	Angus	AN	20	0.338 ± 0.180	0.332 ± 0.158	0.252 ± 0.149	81
36	Holstein	HO	20	0.353 ± 0.174	0.347 ± 0.149	0.264 ± 0.145	71

Number of individuals per breed (N), observed (Ho) and expected (He) heterozygosity, average minor allele frequency (MAF), effective population size (Ne) and standard deviation (SD)

### SNP data and genetic diversity

SNP genotypes were obtained from previous studies [4, 23, 26–32], with the exception of the data for Bulgarian Grey and Turkish Grey animals, which we generated specifically for this study (see Additional file 1: Table S1). In addition, we also used the markers extracted from the high-density genome-wide SNP panel for the British aurochs [23] that were common to the autosomal SNPs mapped on the Illumina BovineSNP50. The merged dataset consisted of 48,637 SNPs. After merging, the SNP data were pruned based on minor allele frequency (MAF) and on missing genotype call rate (--maf 0.05, --geno 0.01) using the PLINK software [33], which resulted in a dataset that included 29,549 SNPs. For each breed, observed (H_o_) and expected (H_e_) heterozygosity and average MAF were estimated using PLINK and trends in effective population size (Ne) based on linkage disequilibrium (LD) were estimated using the SNeP v1.1 software [24].

### Genetic relationship and admixture

To reduce the number of SNPs in LD, we pruned the dataset using the --indep-pairwise flag in PLINK by removing SNPs with an r^2^ higher than 0.1 within 50-SNP windows in 10 steps, thus obtaining a final dataset consisting of 17,096 SNPs (17 K).

To explore the genetic differentiation of the whole dataset, the pairwise genetic relationships were estimated using a matrix of genome-wide identity-by-state (IBS) genetic distances calculated by PLINK and plotted in a multidimensional scaling (MDS) plot. To assess reticulated relationships between populations, pairwise Reynolds genetic distances were estimated with the ARLEQUIN [34] software, and then used to construct a Neighbor-Net graph with SPLITSTREE [35]. The Neighbor-Net graph was built using both the complete dataset and a reduced dataset including only the European taurine breeds and the Guelmoise breed.

ADMIXTURE v 1.2 [36] was used to infer ancestry proportions of *K* ancestral populations using a cross-validation approach (CV = 10) for *K* values ranging from 2 to 23. Then, the R package BITE [37] was used to plot the obtained Q matrix of membership coefficients with the *membercoef.circos* function.

Finally, we used the software TREEMIX [38] to explore genetic relationships and migration events among breeds, by modelling genetic drift at genome-wide polymorphisms. The maximum likelihood dendrogram was generated based on the observed covariance structure between breeds. When breeds did not fit a bifurcating tree, an admixture event was added. This analysis was also performed on the reduced dataset including only the European taurine cattle breeds and the Guelmoise breed. Both analyses were performed using the 29 K (unpruned) dataset with five iterations, allowing migration events from 1 to 10 and taking LD across blocks of 500 contiguous SNPs into account. The most predictive number of migration events was evaluated using the *optM* function in the R package OptM (Fitak RR: an R package to optimize the number of migration edges using threshold models, submitted).

### Approximate Bayesian computation and scenarios of colonization

To ensure that our dataset contained only putatively neutral loci, we used the package *pcadapt* v.4.1.0 [39] to identify and remove candidate markers that were significantly unrelated to population structure, thus conceivably under selection. The best number of principal components to be retained (*K* = 5) was evaluated using the graphical ‘scree plot’ approach [40] and *p*-values of the Mahalanobis distances were used as the test statistics to detect outlier SNPs. The distribution of the observed *p-*values was checked against the expected *p*-values using a Q-Q plot. The *q*-value procedure implemented in the qvalue R package was performed by setting a cut-off of 0.1% as false discovery rate threshold for detecting outlier SNPs that were likely under selection. Thus, 314 SNPs were removed from the dataset. In addition, to reduce the computational burden, the dataset was further pruned for LD using the --indep flag with an *r*^*2*^ threshold of 1.2% resulting in 8440 (8 K) SNPs being retained. To assess if this pruned dataset still contained a sufficient amount of information regarding genetic structure, we performed a principal component analysis (PCA) as implemented in *pcadapt* and compared results to those obtained using the complete dataset.

Possible colonization routes of the Podolian cattle were evaluated in an Approximate Bayesian Computation (ABC) framework using a tree-based classification method as implemented in the DIYABC random forest software [41]. This method uses a supervised machine learning algorithm that allows to classify scenarios and to estimate parameter robustness based on 1000 to 10,000 simulations per scenario.

For this analysis, breeds were grouped based on both MDS and TREEMIX results and two populations per group were selected. This process aimed at avoiding outliers or topological inconsistencies and at assessing whether the outcomes could be related to breed choice rather than reflecting their demographic history. In doing so, we did not consider breeds with strong inbreeding values or low genetic diversity levels, to retain an adequate amount of ancestry information to be incorporated at the coalescence. Simulations were then performed by grouping individuals into five main populations according to the main geographic areas (two indicine breeds from Asia: Gabrali and Gir; two European non-Podolian breeds: Angus and Holstein; two Italian Podolian breeds: Maremmana and Podolica Italiana; two Balkan Podolian: Hungarian Grey and Croatian Podolian; two South-East Podolian: Turkish Grey and Katerini).

**Fig. 1:**
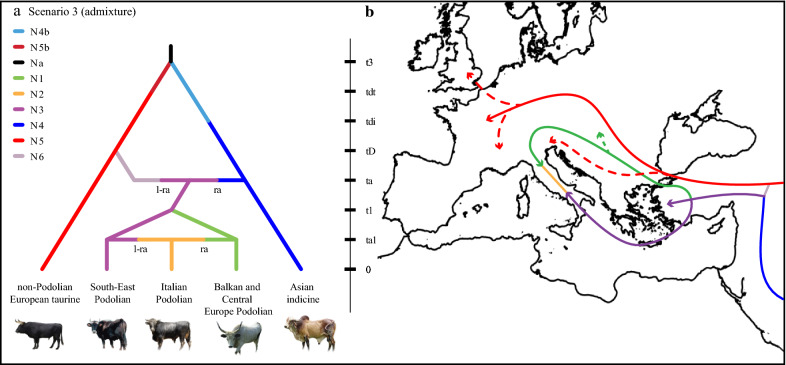
**a** Schematic representation of the most voted scenario of colonization.The model predicts a first separation between taurine and indicine (t3) followed by independent domestication events (tdt and tdi) that took place in the Fertile Crescent and the Indus Valley, respectively. An early non-Podolian taurine migration (tD) occurred before the admixture event between taurine and indicine (ta). This scenario, simulates an admixture event (ta1) between the Balkan and Central Europe Podolian and the South-East Podolian leading to the formation of the Italian Podolian breeds. **b** A geographic map indicating the presumed migration routes as inferred for Scenario 3. Arrows and dotted arrows indicate the assumed or alternative (Mediterrranean or Danube) migration routes

To model our scenarios we assumed that: (i) all extant breeds had a common ancestor which gave rise to indicine and taurine lineages; (ii) the taurine domestication took place in the Fertile Crescent between 3600 and 7800 generations ago [9]; and (iii) the emergence of indicine introgression, which was revealed by analysing ancient DNA, appeared across the Near East not earlier than 4200 YBP [5]. From these known events, we generated two groups of scenarios. For the first set, we modelled taurine cattle as colonizing Europe by two waves of migration, i.e. an early migration that occurred before the indicine introgression of non-Podolian taurine cattle followed by a second migration of Podolian cattle. Conversely, in the second set of scenarios, we assumed that the observed taurine genetic diversity results mainly from a single migration wave that occurred at the onset of the current geological age with genetic replacement of earlier migrations. For each set of models, three different scenarios were designed. Under Scenario 1 (2 waves—Mediterranean route), we tested the appealing hypothesis of an Anatolian origin of the Italian Podolian breeds via sea routes conceivably during the Etruscan period. In Scenario 2 (2 waves—Balkan route), we assumed that, following a terrestrial model of colonization probably via the Danube route, Podolian breeds colonized Italy later by moving southward along land routes. An alternative Scenario 3 (2 waves—admixture) was built under the assumption of an equal contribution of the two routes (Mediterranean and Balkan) followed by admixture events leading to the formation of the Italian Podolian breeds (Fig. 1). The remaining Scenarios 4, 5 and 6 mirror Scenarios 1, 2 and 3 (Mediterranean, Balkan and admixture) except for the position of the non-Podolian taurine which are nested within a single wave [for more details on each scenario (see Additional file 2: Figure S1)].

To have an internal time calibration point, we incorporated a uniform prior distribution, with lower and upper bounds at 3600 and 7800 generations, respectively, to account for the size reduction that occurred during domestication in the Fertile Crescent (see Additional file 3: Table S2) for prior information). To produce a posterior distribution, we simulated 30,000 datasets, and 1000 trees were used to grow a random forest classification. Taking advantage of the model-grouping approach, which allows to evaluate whether or not a particular evolutionary event is of prime importance to explain the observed dataset, we made a first choice of model between the two sets of scenarios (1 wave vs. 2 waves). Subsequently, the choice of the random forest model was used to classify all modelled scenarios. The compatibility between scenarios and/or associated priors and the observed data was assessed using linear discriminant analysis (LDA) as implemented in the DIYABC random forest software.

## Results

### Genetic diversity and population structure

Genetic indices including observed (H_o_) and expected (H_e_) heterozygosity, minor allele frequencies (MAF) and effective population size (Ne) are in Table 1 and Figure S2 (see Additional file 4: Figure S2). The European taurine cattle display higher values for all genetic diversity indices than both the African taurine and Asian indicine breeds. The Podolian breeds show medium to high values of H_o_ and H_e_. Regarding Ne, we observed low values for Asian indicine breeds and some Podolian breeds such as the Calvana, Maremmana, Serbian Podolsko, Croatian Podolian, Bulgarian Grey and Romanian Grey, with the latter being represented, in our dataset, by only four individuals.

**Fig. 2:**
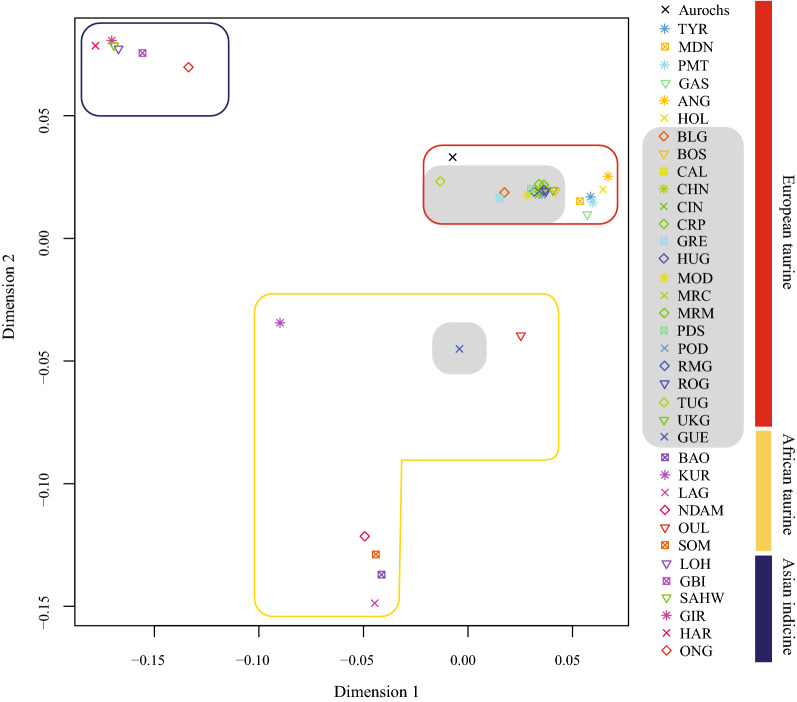
MDS plot showing the genetic relationships among all cattle breeds analysed in this study. The grey colour indicates all the breeds that have been either traditionally acknowledged as of Podolian origin or that display phenotypic traits shared among Podolian breeds. For a full definition of the breeds see Table 1

Genetic differentiation as illustrated in the MDS plot confirms the presence of three principal clusters according to breed geographic origin (Fig. 2). All the Podolian cattle cluster within the group of European taurine breeds but show a slight differentiation with respect to the cosmopolite Angus and Holstein breeds and other taurine breeds such as Modenese, Piedmontese, Tyrolean Grey and Gascon. All Podolian cattle cluster close to each other, with the exception of the Guelmoise which falls middle way between African and European taurine breeds (Fig. 2).

**Fig. 3:**
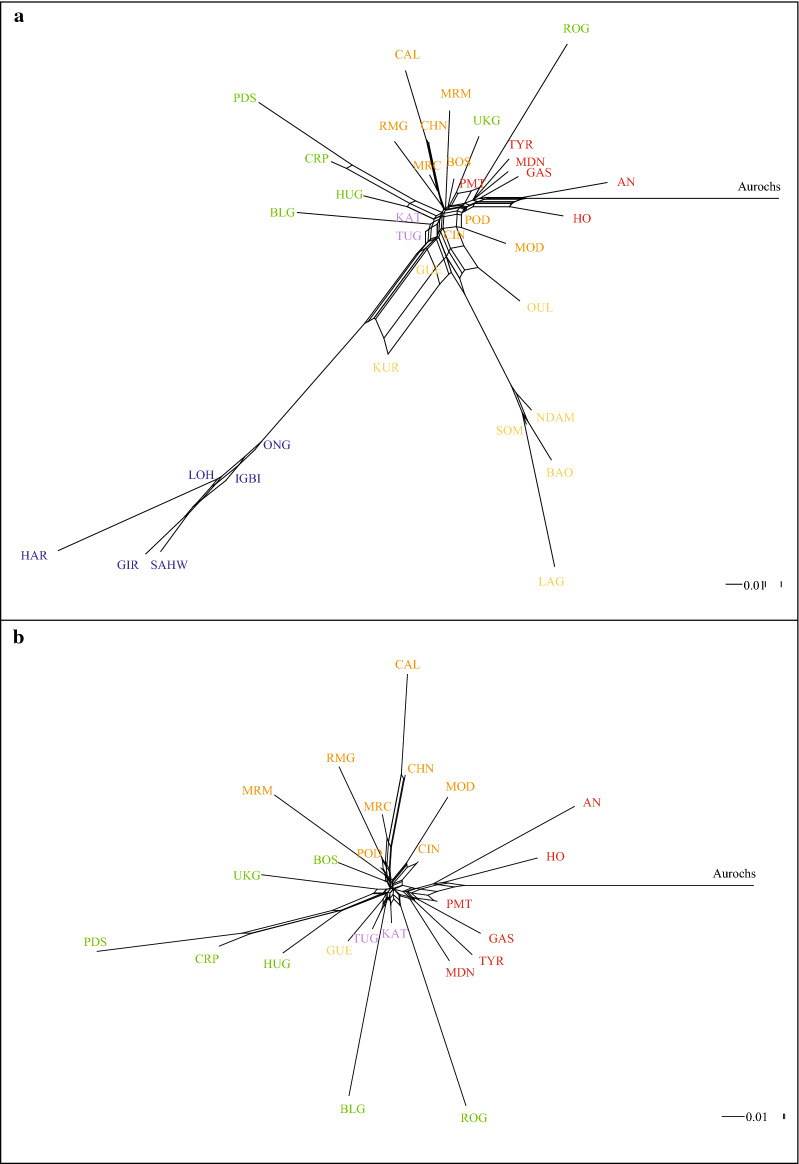
Neighbor-net graph based on Reynolds genetic distances for the whole dataset (**a**) and for the reduced dataset including only European taurine breeds (**b**). Asian indicine (blue), African taurine (yellow), non-Podolian taurine (red), Balkan Podolian (green), Italian Podolian (orange), South-East Mediterranean Podolian (purple), aurochs (black). For a full definition of the breeds see Table 1

**Fig. 4:**
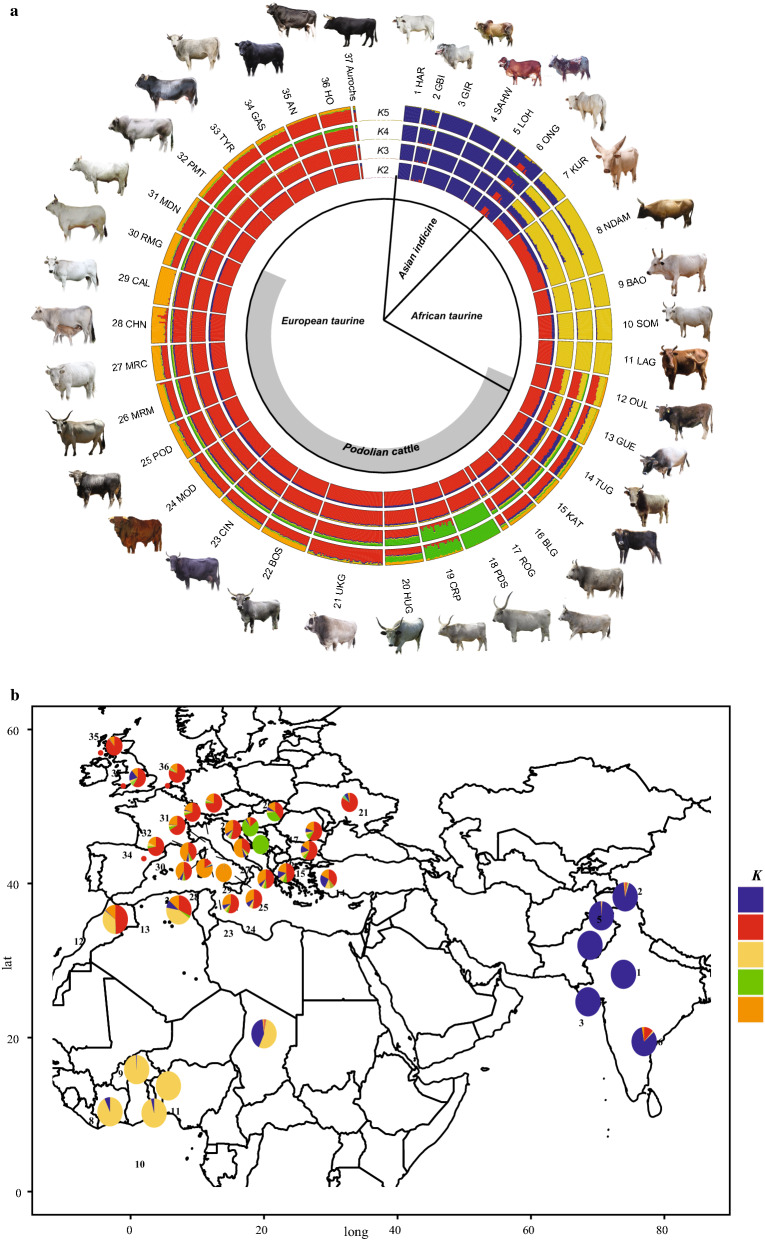
ADMIXTURE analysis results for *K* values ranging from 2 to 5 (**a**) and geographic distribution of the genomic components corresponding to the *K* = 5 resolution (**b**). For a full definition of the breeds see Table 1

The Neighbor-Net graph confirms the MDS results and points out the separation between taurine and indicine breeds (Fig. 3a). The long branches for the African taurine and the indicine cattle and the aurochs reflect the combined effects of ascertainment bias and distinctiveness. This network also separates the Podolian breeds on the basis of genetic origin and/or of their geographical proximity (Fig. 3b). The ADMIXTURE analysis showed that the lowest value of cross-validation error (CV) occurs at *K* = 23, but the largest reduction in CV error occurs between *K* = 2 and *K* = 5 (see Additional file 5: Figure S3). The results of the population structure analysis consistently confirm the remarkable subdivisions among the breeds and the main genetic groups observed in the MDS (Fig. 4a). The graph shows the separation between *B. t. taurus* and *B. t. indicus* at *K* = 2 and the separation of African taurine at *K* = 3,. Interestingly, whereas the African taurine component can be detected only in a few other breeds outside Africa and with a low percentage (Katerini and Turkish Grey), the indicine component shows a higher penetration, reflecting an East–West cline which has almost completely disappeared in the non-Podolian European taurine breeds such as Holstein, Angus, Piedmontese, Tyrolean Grey, Gascon and Modenese (Fig. 4b). At *K* = 4, the Serbian Podolsko shows a separate genomic component, which is present at varying degrees in almost all the taurine breeds, except the African taurine and indicine breeds. At *K* = 5, the graph highlights a component that is shared by the Calvana and Chianina breeds and appears to be dominant in several Italian Podolian breeds but is very low or even absent in Central European, Balkans and Mediterranean Podolian breeds. When *K* increases from 6 to 23, breeds are progressively assigned to separate clusters but with some exceptions. For example, a shared component (from *K* = 11 to 22) is revealed among the Gascon, Tyrolean Grey, Piedmontese and Modenese as well as among the Asian indicine and African taurine breeds (see Additional file 6: Figure S4). Conversely, the South and Eastern Mediterranean Podolian breeds (Romanian Grey, Katerini, Turkish Grey and Guelmoise) show less distinct clusters with a mosaic of different genetic components including indicine, African taurine and European taurine contributions. In addition, some Italian and Balkan Podolian breeds show high levels of genetic admixture such as the Boskarin, Podolica Italiana and Marchigiana.

**Fig. 5:**
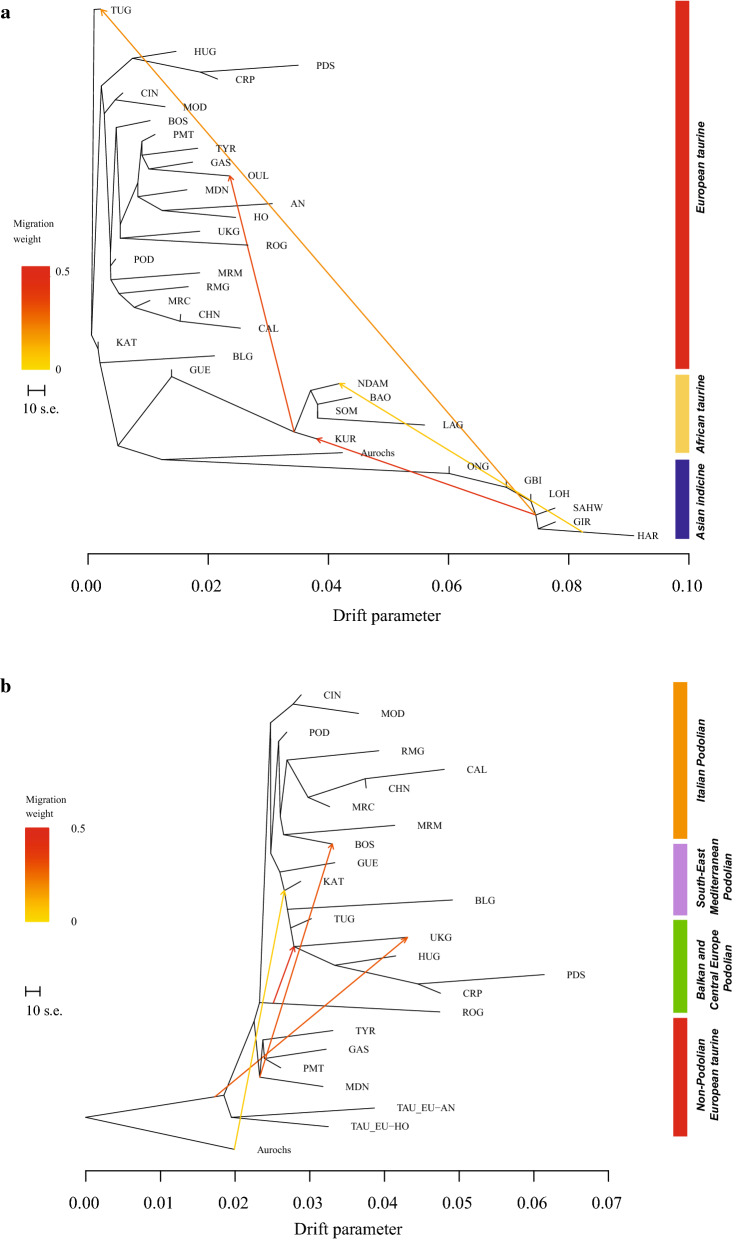
Maximum likelihood trees as inferred in TREEMIX using the whole dataset (**a**) and the Podolian dataset (**b**). Arrows indicate migration edges coloured according to the proportional ancestry received from the donor population. For a full definition of the breeds see Table 1

We used the TREEMIX software to model both population splits and gene flow using the whole dataset (Fig. 5a) and a reduced dataset including only the European taurine breeds (Fig. 5b). The results using the whole dataset basically confirm this genetic structuring, with indicine and African taurine breeds forming two basal groups and the sample of aurochs positioned between them. Within the European taurine breeds, South-Eastern breeds (Katerini, Turkish Grey, Bulgarian Grey and Guelmoise) diverge first, with the Guelmoise being more related to the African taurine breeds. The Podolian breeds from Italy (Maremmana, Marchigiana, Romagnola, Podolica Italiana, Chianina and Calvana) also form a distinct cluster whereas the two Sicilian breeds (Modicana and Cinisara) appear more related to some Balkan Podolian breeds (Serbian Podolsko, Croatian Podolian and Hungarian Grey), which form a distinct group. The remaining Podolian cattle (Boskarin, Ukrainian and Romanian Grey) cluster at a basal position with respect to all other European taurine breeds, with the Ukrainian and Romanian Grey breeds showing a close relationship. When migration edges were modelled in the analysis, the *optM* function indicated four migration events as the most reliable number for the whole dataset (see Additional file 7: Figure S5). Stronger migration weights were estimated between some indicine breeds (Sahiwal, Gir and Hariana) with both African Kuri and Turkish Grey, and between all African taurine and the Oulmès Zaer from Morocco, whereas a weaker migration edge was estimated between the Hariana and the N'Dama breeds (Fig. 5a).

The tree-based underlying graph shows that the genetic relationships among the European taurine breeds reflect their geographic origin. The Italian Podolian breeds including the Sicilian breeds (Modicana and Cinisara) are highly related, whereas the South-Eastern Mediterranean Podolian and the Balkan and Central European Podolian breeds form a single cluster, with genetic relationships consistent with their geographic native area. All other non-Podolian taurine breeds are at a basal position of the tree (Fig. 5b). The *optM* function supported four migration events (see Additional file 8: Figure S6). Three strong migration weights are observed between the Romanian Grey and the Balkan breeds (Ukrainian Grey, Hungarian Grey, Serbian Podolsko and Croatian Podolian), between the base of the tree and the Ukrainian Grey, and between the European non-Podolian breeds and the Boskarin breed. Interestingly, a weaker migration weight is also detected between the British aurochs and the base of a group including Eastern Mediterranean Podolian, Balkans and Central Europe Podolian breeds (Fig. 5b).

### ABC framework and scenarios of colonization

The 8 K dataset carried a level of information on the genetic structure of the analysed breeds that was similar to that of the larger dataset (see Additional file 9: Figure S7), which indicates that it was well suited to carry out ABC analysis without a significant loss of informativeness.

In the LDA projection, observed data were within the simulated dataset (see Additional file 10: Figure S8). By comparing colonization scenarios, we found more support for set 1 (696 votes out of 1000 RF-trees, posterior probability = 0.82), which indicates that European cattle have followed at least two waves of colonization. Among all the competing historical scenarios of colonization in the ABC-based analysis, the highest classification vote was assigned to Scenario 3, which assumes an admixture event that gave rise to the Italian Podolian (316 votes out of 1000 RF-trees, posterior probability = 0.49). This scenario predicts that following the separation of the non-Podolian taurine breeds during an early colonization wave, an admixture event occurred between extant taurine and indicine cattle. Finally, a more recent migration through both terrestrial (Balkans) and sea (Mediterranean) routes followed by successive reconnection and admixture was modelled in order to explain the origin of the Italian Podolian breeds. All posterior parameter estimates with relative 95% confidence intervals are in Table 2.

**Table 2:** Parameter estimates and associated 95% confidence intervals defined by the 0.05 and 0.95 quantiles (Q) of the posterior distribution for the best supported Scenario 3

Parameter	Median	Q_0.05_	Q_0.95_
N1	3332.69	749.13	11,558.50
N2	2967.79	312.98	14,148.00
N3	11,984.00	4075.93	29,968.50
N4	6565.52	2446.93	17,398.00
N4b	30,831.20	10,869.50	48,592.00
N5	21,373.00	9341.48	42,420.80
N5b	34,525.40	18,558.90	48,333.50
ta1	790.00	161.00	2053.10
t1	938.00	259.11	2690.00
ta	1874.90	657.30	4320.35
tD	2593.00	913.86	5439.00
tdi	4026.40	1751.91	6684.38
tdt	5433.79	3734.90	7433.83

Number of individuals for effective population size parameter (N) and number of generations (t) are reported

## Discussion

The evolutionary history of European taurine cattle is complex and dominated by repeated introgression events with both local aurochs and livestock that followed human movements and trades after the Neolithic expansion [8, 11]. The origin of Podolian cattle and their diffusion to southern Europe is still debated among geneticists [23], therefore we used SNP data with the specific aim to contribute to this controversy.

Our results indicated that almost all the breeds that we refer to as Podolian, share a common evolutionary history, with a large part of their level of relatedness explained by their geographic origin (Fig. 4b). Moreover, contrary to other European taurine breeds, they all share a small portion of indicine component (Fig. 4a). The presence of indicine introgression has been repeatedly reported in several South European breeds, including the Balkan Busha and Italian Podolian (Romagnola, Marchigiana, Maremmana, Chianina and Podolica Italiana) [4, 9, 23–42]. Two main hypotheses have been advanced to explain this pattern. On the one hand, it has been interpreted as the outcome of introgression events that occurred in South-West Asia and subsequently spread to South-East Europe during human migrations, pastoralism and trade [4, 42]. On the other hand, a more complex introgression pattern from diverse wild populations emerged from the analysis of the DNA of the first ancient aurochs [43]. In this scenario, wild aurochsen that have an incomplete lineage sorting introgressed Neolithic taurine. Such an event cannot be completely ruled out when considering the recent separation of *Bos primigenius primigenius* and *Bos primigenius nomadicus* (ancestors of the current taurine and indicine subspecies). Indeed, 250,000–300,000 years is a rather short time to allow complete genome segregation as confirmed by the presence in the British wild aurochs of the same genomic component that is found at a high frequency in the modern indicine breeds (Fig. 4a). Although this introgression pattern has been highlighted in some Northern European cattle [43], additional local hybridizations between wild aurochs and European cattle, especially Italian Podolian and Balkan breeds, might have been more frequent and widespread than expected [11, 16]. This is also in line with the migration events highlighted by the TREEMIX analysis which indicates admixture between the aurochs and the basal node of a group of Podolian breeds including the Katerini, Bulgarian Grey, Turkish Grey, Hungarian Grey, Ukrainan Grey, Podolsko Serbian and Croatian Podolian (Fig. 5b). Analysis of additional samples of aurochs, from Northern Europe, Balkan and Italy, is recommended to disentangle the nature and the origin of the observed genomic patterns.

### Genomic relationships of Podolian cattle

Previous studies have explored the genetic variability of a limited number of Podolian breeds, mostly by using mtDNA [8, 20, 23]. Here, we present a comprehensive study that aimed at reconstructing a general picture of the genomic diversity of these breeds. Podolian cattle and more generally local breeds, represent an important resource not only for traditional farming and products, but also because they might harbour a relevant reservoir of genetic diversity which can contribute to future traits of interest [44]. Our results confirm that most Podolian breeds have a rather high level of heterozygosity (Table 1) and (see Additional file 4: Figure S2). It is interesting to note that the Podolian breeds that show lower values of genetic diversity (Podolsko Serbian, Croatian Podolian and Calvana) are also those for which low values of both nucleotide and haplotype diversity were previously found at the mtDNA level [20]. On the contrary, our MDS plot showed a different clustering compared to the principal component analysis of mtDNA performed by Di Lorenzo et al. [20]. Whereas the mitochondrial signature suggested a close relationship, our genomic data indicated a marked genetic difference between the Turkish Grey and some Italian Podolian breeds (Maremmana, Marchigiana Chianina, Podolica Italiana and Romagnola) (Figs. 2, 3). The presence of such a mito-nuclear discordance is probably due to the different inheritance patterns of the two genomes suggesting a possible role of a sex-biased introgression [5, 45].

Based on the MDS plot, the Podolian cattle cluster closely together with the exception of the Guelmoise breed, which falls middle way between the European and African taurine breeds (Fig. 2). Although the Guelmoise breed shares some morphological traits with other Podolian breeds, such as coat color, our results seem to question the Podolian origin of this breed, as already suggested by its admixed origin and strong genetic similarity to Tunisian cattle pointed out by Ben Jemaa et al*.* [29]. Moreover, our results contrast with those of the MDS plot reported by Zsolnai et al*.* [46] that showed that Hungarian Grey clustered independently from the other European Podolian cattle breeds.

The genetic complexity of Podolian cattle is also evident in the ADMIXTURE analysis, which showed admixture in some Podolian breeds and distinctiveness in other ones (see Additional file 5: Figure S3). In particular at low *K* values, we observed an early separation of the Balkan (*K* = 4) and Italian (*K* = 5) Podolian breeds (Fig. 4a). Such a partition is also supported by the TREEMIX graph (Fig. 5b), which indicates an agreement between clustering and geographic origin. Podolian cattle from the Balkans and Central Europe cluster together, except for the Romanian Grey breed. This behaviour is probably due to the small sample size of this breed (four individuals), which implies caution in the interpretation of its genetic relationships, and also when considering the strong migration edge that connects Romanian Grey the other Balkan breeds which may account for their common genetic origin (Fig. 5b). Among the Balkan breeds, the Podolsko Serbian, Croatian Podolian and Hungarian Grey breeds are the most differentiated, which confirms the genetic differentiation of the Hungarian Grey breed with respect to other Podolian and non-Podolian European taurine found by Zsolnai et al*.* [46]. The observed distinctness of these Balkan breeds (including the Romanian Grey), might be the result of a strong genetic drift, as suggested by the low values obtained for the genetic diversity indices and effective population size (Table 1) and (see Additional file 4: Figure S2) and the long branches in the TREEMIX graphs (Fig. 5).

Our results show that the Boskarin (Istrian cattle) breed is genetically more similar to the Italian Podolian breeds, in particular with Maremmana and Podolica Italiana, than to the Balkan Podolian breeds, which confirms a previous result obtained with microsatellite markers [47] and is not surprising considering that Istria was Italian until 1947 and represented an important trading point between the two Adriatic shores. We also found some genetic relationships between the Boskarin and other non-Podolian breeds from Northern Italy and Austria, as highlighted by the migration edges in the TREEMIX analysis (Fig. 5b).

Both ADMIXTURE and TREEMIX analyses converged in indicating a close relationship among the Italian Podolian breeds (Figs. 4, 5). Although at* K* = 5, the first breeds that separated were Calvana and Chianina, the ADMIXTURE analysis showed the extensive occurrence of a shared genetic component among the Italian Podolian breeds until *K* = 10. The divergence of other Italian Podolian breeds appeared at higher values of *K*, such as for Romangnola (*K* = 14) and Modicana (*K* = 15). Branch lengths and genetic indices seem to suggest that, in particular, the Calvana breed is under strong genetic drift and has a low genetic variability. Furthermore, the tree-based graph reconstruction places the two Sicilian breeds at a basal position with respect to all the other Italian Podolian breeds (Fig. 5), with Modicana displaying lower values of heterozygosity and Ne than Cinisara. These results confirm the Podolian origin of the two Sicilian breeds, as previous reported [22, 27].

### Demographic scenario of colonization

The cradles of cattle genetic diversity are the main centres of domestication in the Fertile Crescent and the Indus Valley. From these regions, cattle accompanied the Neolithic expansion of humans and agriculture and colonized the world following both terrestrial and sea routes, as the two earliest European colonization routes via Mediterranean coasts and along the Danube as suggested by archaeological findings [48, 49]. According to recent findings, the indicine introgression into European taurine cattle is dated no earlier than 4200 YBP [5] and these routes might have witnessed different waves of European taurine diffusion. Our ABC-RF analysis strongly supports a colonization model that includes two principal migrations. An early Neolithic colonization, with no traces of indicine components, of the ancestor of present-day European non-Podolian taurine cattle, and a more recent wave, of the ancestor of the Podolian breeds. Interestingly, the inferred scenario of colonization mirrors the secondary wave of migration, which is thought to have introduced wool-type sheep into Europe that have replaced most of the original genetic stock of hair-type sheep [50, 51].

Our coalescence estimates point to a first split between Podolian and non-Podolian taurine cattle that occurred approximately 6482 YBP (95% CI 2284–13,597 YBP; when considering a generation time for cattle of 2.5 years), which is a plausible time window corresponding to a first Early Neolithic farmer expansion. From Anatolia, the human population expanded through two well-defined streams, along the Danube (Balkan Neolithic, ~ 6000 BC) with several waves of migrants into this region and along the Mediterranean coast [52, 53]. Such evidence of separate colonization routes has also been highlighted in other livestock species [54]. Our estimate of the time of admixture, i.e. 4687 YBP (95% CI 1643–10,800 YBP), between taurine and indicine cattle is consistent with that obtained from the analysis of ancient DNA data [5]. However, these estimates should be considered with caution because they are difficult to infer without bias [55]. First of all, SNP panels suffer from ascertainment bias, which can lead to underestimate the genetic variability of some breeds. In addition, we used an average cattle generation time of 2.5 years, which is likely an approximation. Finally, considering the timescales in the models, incomplete lineage sorting may cause divergence to be rooted more deeply than expected. In spite of these considerations, our estimates of the confidence intervals fall within plausible time windows, which parallel previous genetic, historical and archaeological evidences.

Among the competing historical models, we found support for the admixed origin of the Italian Podolian breeds with a contribution of both Balkan and South-East Podolian gene pools. This result suggests that both the Danube (Balkans) and sea (Mediterranean) routes have played a central role in this secondary migration. In this context, the date of our estimates of the time of admixture is about 1975 YBP (95% CI 402–5132 YBP). Although an Anatolian origin of the Etruscans is still controversial and seems to be denied by the analysis of ancient human DNA [56], a sea route arrival due to the increased commercial trades during the post Etruscan and the Roman periods cannot be completely ruled out. These results might also explain the higher genetic diversity observed in both autosomal DNA and mtDNA data from many Italian cattle [24]. This genetic diversity may have been further increased by occasional hybridization events with local aurochs’ populations, as suggested by Achilli et al. [15]. Since the presence of the rare mitochondrial R lineage in some Italian Podolian breeds (Romagnola, Marchigiana and Cinisara) indicates a matrilinear introgression from local wild aurochs in the autochthonous Italian cattle genomes, the occurrence of a parallel male-mediated contribution, similar to that already observed in many cattle worldwide [45, 57], cannot be ruled out. Thus, further studies based on paleogenomics are needed to better address the dynamics of the expansion of Neolithic cattle and to evaluate the magnitude of the wild aurochs introgression into Italian and European cattle.

## Conclusions

In this study, we used a comprehensive dataset including most of the autochthonous cattle breeds belonging to the so-called Podolian trunk to shed light into the origin and diversification of this group of European cattle. Podolian cattle are a good model to investigate historical routes of colonization in Europe, since they share ancestral traits pointing at a common origin. They also encompass an important fraction of the diversity of the European autochthonous breeds, which represents a fundamental resource and income for traditional farming thanks to local products. Considering the mtDNA signature, there is growing evidence of a decrease in genetic diversity as the distance to the domestication centre increases. However, several local breeds especially from the Italian Peninsula, show levels of genetic diversity that are similar to those observed in autochthonous breeds from the domestication centre, and intriguing explanations have been advanced to disclose this pattern. Our genomic analysis is in line with such a scenario that indicates a notable geographic cline from South-Western Asia to Europe. Furthermore, we also find a marked genomic diversity that localizes within the Italian Peninsula. The presented ABC framework allowed us to test different scenarios of colonization that highlight a complex evolutionary history of European taurine cattle. According to our coalescent and demographic reconstruction, European taurine experienced two principal migration waves, i.e. an early migration which gave rise to the non-Podolian taurine breeds followed by a secondary migration, which was probably prompted by increasing trade that favoured the diffusion of European Podolian. In addition, the most well-supported scenario suggests admixture events as an important driver in shaping the genetic diversity of Podolian cattle, which partially explains the marked genomic diversity observed in the Italian Peninsula. Finally, the role of hybridization with local aurochs’ populations still remains a pending question that needs further investigation.

## Supplementary Information

**Additional file 1: Table S1.** Name of the breeds, breed codes, sample size (N), sub-species, continent and geographic origin, and source of genotyping data.

**Additional file 2: Figure S1.** All modelled scenarios for colonization tested in the ABC framework. Description: In all the tested scenarios, we assumed that taurine and indicine cattle separated first. Subsequent reduction in effective population size was modelled to take the two independent domestication events that occurred in the Fertile Crescent and the Indus Valley into account. From these known evolutionary events, two sets of scenarios were built. The first three scenarios mirror to two different waves of migration, an early Neolithic migration involving non-Podolian taurine cattle and a secondary migration involving Podolian cattle after their genetic admixture with indicine cattle that occurred in South-Western Asia. On the opposite, the other three scenarios reflect a single taurine diffusion that occurred after the admixture event between indicine and taurine. Within each set of scenarios, we drew different hypotheses of colonization. A Mediterranean route (Scenarios 1 and 4) in which the Italian Podolian breeds mainly derived from the South-Eastern Mediterranean region and introduced via sea trade. To model this scenario, we assumed that from an ancestral population of size N3 located in the South-Eastern Mediterranean region, a first colonization prompted the split between the Balkan and Central Europe breeds, while a subsequent split, driven by sea routes, led to the formation of the Italian Podolian breeds. In this latter separation, we incorporated a reduction in effective population size to accommodate a founder effect as expected when an population introduced by sea trade starts spreading from few individuals. A Balkan route (Scenario 2 and 5) in which we assumed a terrestrial model of colonization, that therefore we modelled from an ancestral population of size N3 located in South-Eastern Mediterranean, a first split gave rise to Balkan and Continental Podolian breeds while a subsequent divergence event led to the origin of the Podolian cattle in Italy. In this model, we do not constrain priors on effective population size in order to simulate a long-term dispersal as a consequence of a terrestrial expansion. Finally, the admixture scenario simulates an equal contribution of the two routes followed by admixture events leading to the formation of the Italian Podolian breeds.

**Additional file 3: Table S2.** Set of priors used to model the scenarios in the ABC framework.

**Additional file 4: Figure S2.** Genetic diversity indices: observed and expected heterozygosity (H_o_ and H_e_), effective population size (Ne) and minor allele frequencies (MAF) calculated for each breed. Asian indicine (blue), African taurine (yellow), European Podolian (orange), European non-Podolian (red).

**Additional file 5: Figure S3.** Cross-validation plot of the admixture analysis for all values of *K* (number of clusters) ranging from 2 to 23.

**Additional file 6: Figure S4.** Admixture analysis plot in a circular fashion with all values of *K* (number of clusters) ranging from 2 to 23.

**Additional file 7: Figure S5.** Increment in the log likelihood for the complete dataset for all tested migration events, calculated by using the *optM* function in the R package OptM.

**Additional file 8: Figure S6.** Increment in the log likelihood for the reduced (European taurine and the Guelmoise) dataset for all tested migration events, calculated by using the *optM* function in the R package OptM.

**Additional file 9: Figure S7.** Principal component analysis (PCA) for the two datasets used (17 K and 8 K).

**Additional file 10: Figure S8.** Projection on a single LDA axis in the model-grouping approach (a) and on the first two LDA axes in the six scenarios separately (b).

## Data Availability

The data that support the findings of this study are available on request from the corresponding author.
