# Supplementary material for: On the origin and diversification of Podolian cattle breeds: testing scenarios of European colonization using genome-wide SNP data
Source: Genet Sel Evol. 2021 Jun 2;53:48. doi: 10.1186/s12711-021-00639-w (PMC8173809; doi:10.1186/s12711-021-00639-w)
Supplement: Supplementary file 1 — Additional file 1: Table S1. Name of the breeds, breed codes, sample size (N), sub-species, continent and geographic origin, and source of genotyping data. [file 12711_2021_639_MOESM1_ESM.doc]

**Table S1** Name of the breeds, breed codes, sample size (N), sub-species, continent and geographic origin, and source of genotyping data.

| **Breed** | **Code** | **N** | **Subspecies** | **Continent** | **Geographic Origin** | **Data Source** |
| --- | --- | --- | --- | --- | --- | --- |
| Baoule | BAO | 20 | *Bos t. taurus* | Africa | Burkina Faso | Gautier *et al*. 2010 |
| Kuri | KUR | 20 | *Bos t. taurus* | Africa | Chad | Gautier *et al*. 2010 |
| Lagune | LAG | 20 | *Bos t. taurus* | Africa | Benin | Gautier *et al*. 2010 |
| N'Dama | NDAM | 51 | *Bos t. taurus* | Africa | Ivory Coast, Africa_Burkina Faso | Gautier *et al*. 2010 |
| Oulmès Zaer | OUL | 19 | *Bos t. taurus* | Africa | Morocco | Gautier *et al*. 2010 |
| Somba | SOM | 20 | *Bos t. taurus* | Africa | Togo | Gautier *et al*. 2010 |
| Lohani | LOH | 10 | *Bos t. indicus* | Asia | Northwest Pakistan | Decker et al., 2014 |
| Sahiwal | SAHW | 17 | *Bos t. indicus* | Asia | Punjab, Pakistan | Decker et al., 2014 |
| Gir | GIR | 20 | Bos t. indicus | Asia | Gujerat, India | Decker et al., 2014 |
| Hariana | HAR | 10 | *Bos t. indicus* | Asia | Haryana plains, India | Decker et al., 2014 |
| Gabrali | GBI | 10 | *Bos t. indicus* | Asia | Khyber Pakhtun Khwa, Pakistan | Decker et al., 2014 |
| Ongole Grade | ONG | 20 | *Bos t. indicus* | Asia | Andhra Pradesh, India | Decker et al., 2014 |
| Angus | AN | 20 | *Bos t. taurus* | Europe | Scotland | Decker et al., 2014 |
| Holstein | HO | 20 | *Bos t. taurus* | Europe | Europe | Decker et al., 2014 |
| Guelmoise | GUE | 24 | *Bos t. taurus* | Africa | Algeria | Ben Jemaa et al., 2018 |
| Bulgarian Grey | BLG | 20 | *Bos t. taurus* | Europe (Balkans) | Bulgary | This study |
| Istrian cattle (Boskarin) | BOS | 30 | *Bos t. taurus* | Europe (Balkans) | Istria, Croatia | Ramljak et al., 2018 |
| Calvana | CAL | 24 | *Bos t. taurus* | Europe (Italy) | Mugello, Italy | Mastrangelo et al., 2018 |
| Chianina | CHN | 23 | *Bos t. taurus* | Europe  (Italy) | Val di Chiana, Italy | Mastrangelo et al., 2018 |
| Cinisara | CIN | 30 | *Bos t. taurus* | Europe (Italy) | Cinisi, Italy | Mastrangelo et al., 2014 |
| Croatian Podolian | CRP | 24 | *Bos t. taurus* | Europe (Balkans) | Split, Croatia | Ramljak et al., 2018 |
| Katerini | KAT | 19 | *Bos t. taurus* | Europe | Thrace, Greece | Flori et al., 2019 |
| Tyrolean Grey | TYR | 24 | *Bos t. taurus* | Europe | Tyrol, Austria | Ramljak et al., 2018 |
| Hungarian Grey | HUG | 24 | *Bos t. taurus* | Europe (Balkans) | Pushka, Hungary | This study |
| Modenese | MDN | 24 | *Bos t. taurus* | Europe | Modena, Italy | Mastrangelo et al., 2018 |
| Modicana | MOD | 30 | *Bos t. taurus* | Europe (Italy) | Modica, Italy | Mastrangelo et al., 2014 |
| Marchigiana | MRC | 22 | *Bos t. taurus* | Europe (Italy) | Macerata, Italy | Mastrangelo et al., 2018 |
| Maremmana | MRM | 25 | *Bos t. taurus* | Europe (Italy) | Tarquinia, Italy | Mastrangelo et al., 2018 |
| Serbian Podolsko | PDS | 24 | *Bos t. taurus* | Europe (Balkans) | Belgrade, Serbia | This study |
| Piedmontese | PMT | 21 | *Bos t. taurus* | Europe | Carrù, Italy | Mastrangelo et al., 2018 |
| Gascon | GAS | 20 | *Bos t. taurus* | Europe | Southwest France | Gautier *et al*. 2010 |
| Podolica Italiana | POD | 24 | *Bos t. taurus* | Europe (Italy) | Potenza, Italy | Mastrangelo et al., 2018 |
| Romagnola | RMG | 21 | *Bos t. taurus* | Europe (Italy) | Ferrara, Italy | Mastrangelo et al., 2018 |
| Romanian Grey | ROG | 4 | *Bos t. taurus* | Europe (Balkans) | Bucarest, Romania | Upadhyay et al., 2017 |
| Turkish Grey | TUG | 24 | *Bos t. taurus* | Europe | Anatolia, Turky | This study |
| Ukrainian Grey | UKG | 48 | *Bos t. taurus* | Europe  (Balkans) | Kiev, Ukraine | Yurchenko et al., 2017 |
