# Supplementary figures and images for: On the origin and diversification of Podolian cattle breeds: testing scenarios of European colonization using genome-wide SNP data
Source: Genet Sel Evol. 2021 Jun 2;53:48. doi: 10.1186/s12711-021-00639-w (PMC8173809; doi:10.1186/s12711-021-00639-w)

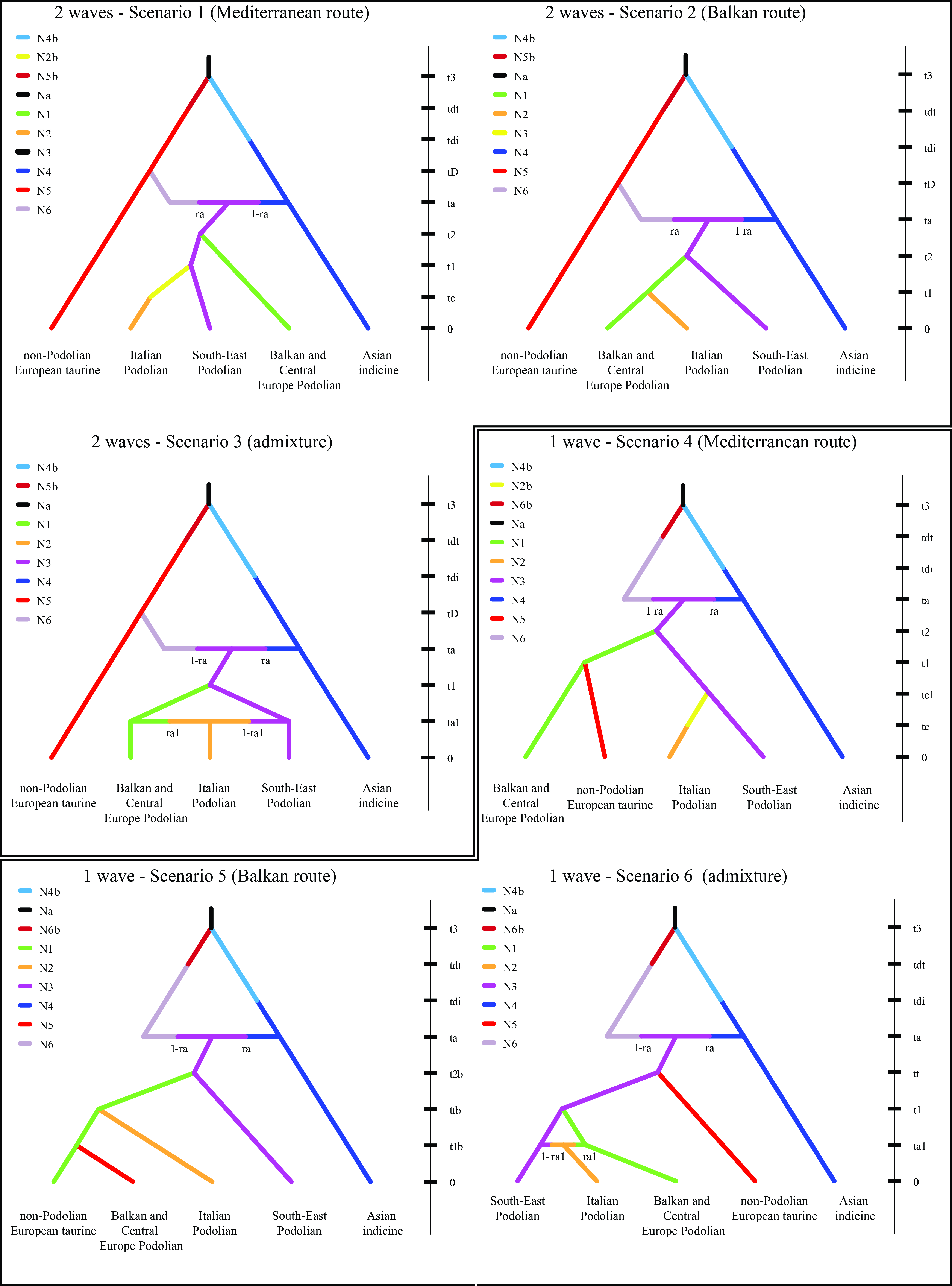

Supplement: Supplementary file 2 — Additional file 2: Figure S1. All modelled scenarios for colonization tested in the ABC framework. Description: In all the tested scenarios, we assumed that taurine and indicine cattle separated first. Subsequent reduction in effective population size was modelled to take the two independent domestication events that occurred in the Fertile Crescent and the Indus Valley into account. From these known evolutionary events, two sets of scenarios were built. The first three scenarios mirror to two different waves of migration, an early Neolithic migration involving non-Podolian taurine cattle and a secondary migration involving Podolian cattle after their genetic admixture with indicine cattle that occurred in South-Western Asia. On the opposite, the other three scenarios reflect a single taurine diffusion that occurred after the admixture event between indicine and taurine. Within each set of scenarios, we drew different hypotheses of colonization. A Mediterranean route (Scenarios 1 and 4) in which the Italian Podolian breeds mainly derived from the South-Eastern Mediterranean region and introduced via sea trade. To model this scenario, we assumed that from an ancestral population of size N3 located in the South-Eastern Mediterranean region, a first colonization prompted the split between the Balkan and Central Europe breeds, while a subsequent split, driven by sea routes, led to the formation of the Italian Podolian breeds. In this latter separation, we incorporated a reduction in effective population size to accommodate a founder effect as expected when an population introduced by sea trade starts spreading from few individuals. A Balkan route (Scenario 2 and 5) in which we assumed a terrestrial model of colonization, that therefore we modelled from an ancestral population of size N3 located in South-Eastern Mediterranean, a first split gave rise to Balkan and Continental Podolian breeds while a subsequent divergence event led to the origin of the Podolian cattle in [file 12711_2021_639_MOESM2_ESM.jpg]

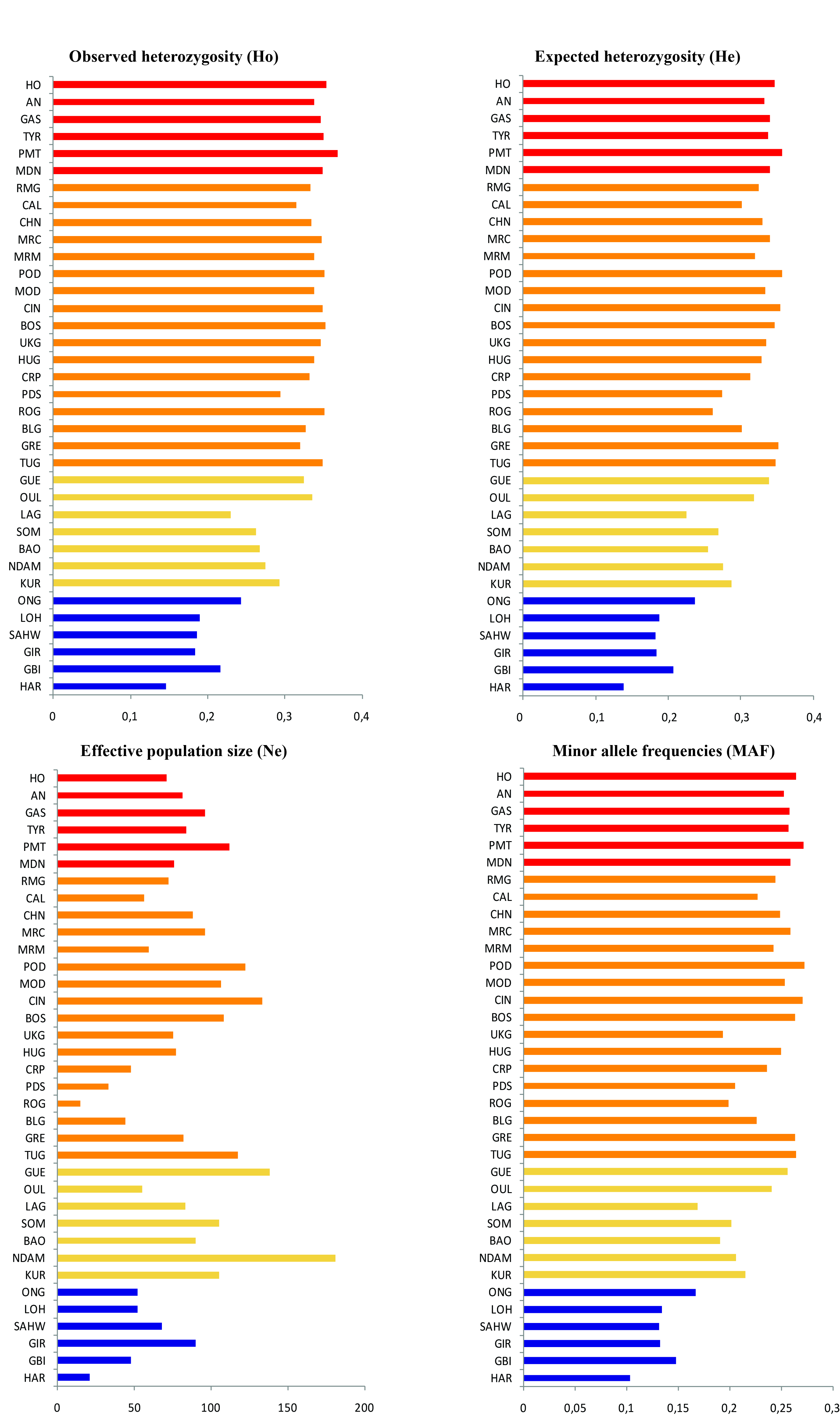

Supplement: Supplementary file 4 — Additional file 4: Figure S2. Genetic diversity indices: observed and expected heterozygosity (Ho and He), effective population size (Ne) and minor allele frequencies (MAF) calculated for each breed. Asian indicine (blue), African taurine (yellow), European Podolian (orange), European non-Podolian (red). [file 12711_2021_639_MOESM4_ESM.jpg]

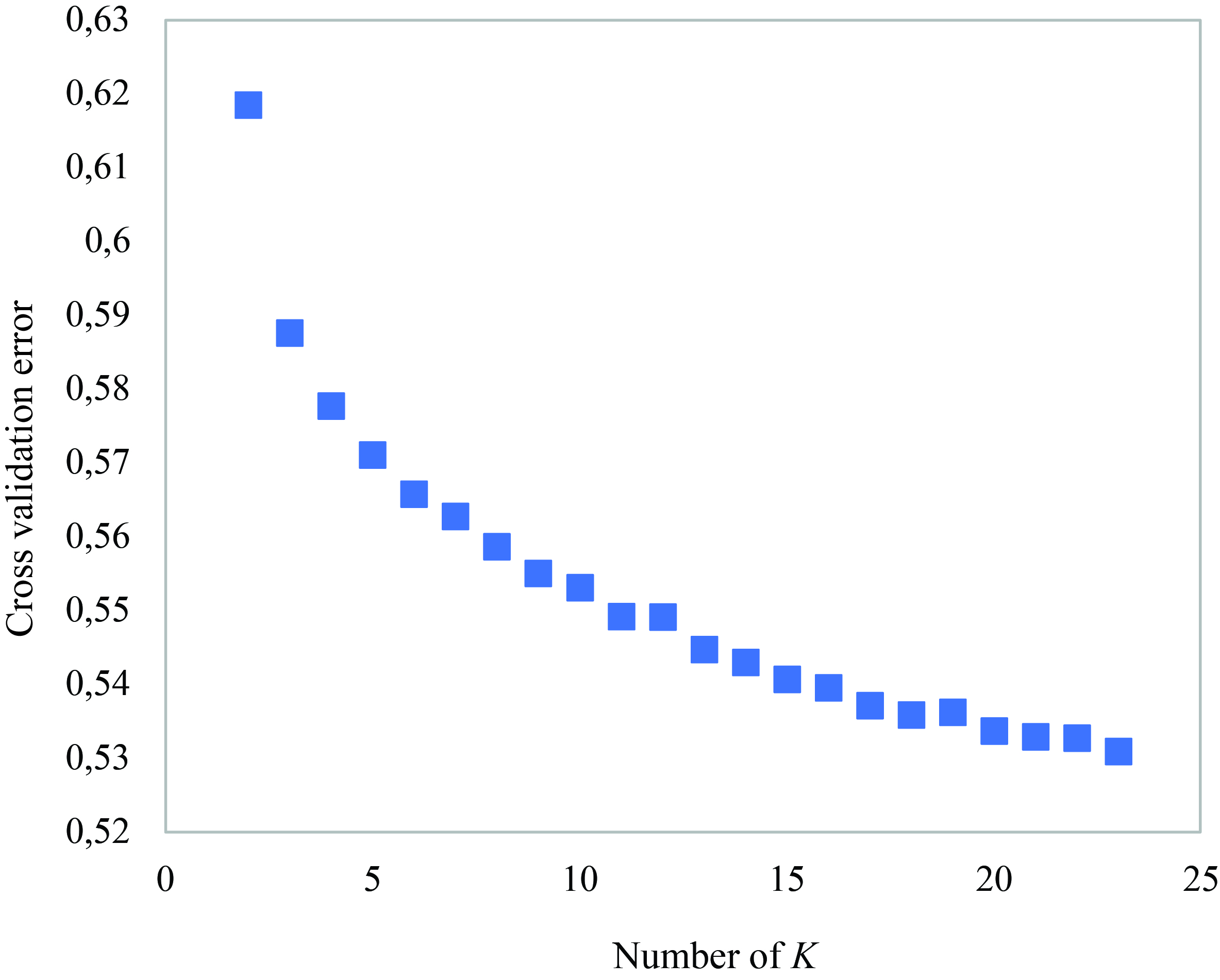

Supplement: Supplementary file 5 — Additional file 5: Figure S3. Cross-validation plot of the admixture analysis for all values of K (number of clusters) ranging from 2 to 23. [file 12711_2021_639_MOESM5_ESM.jpg]

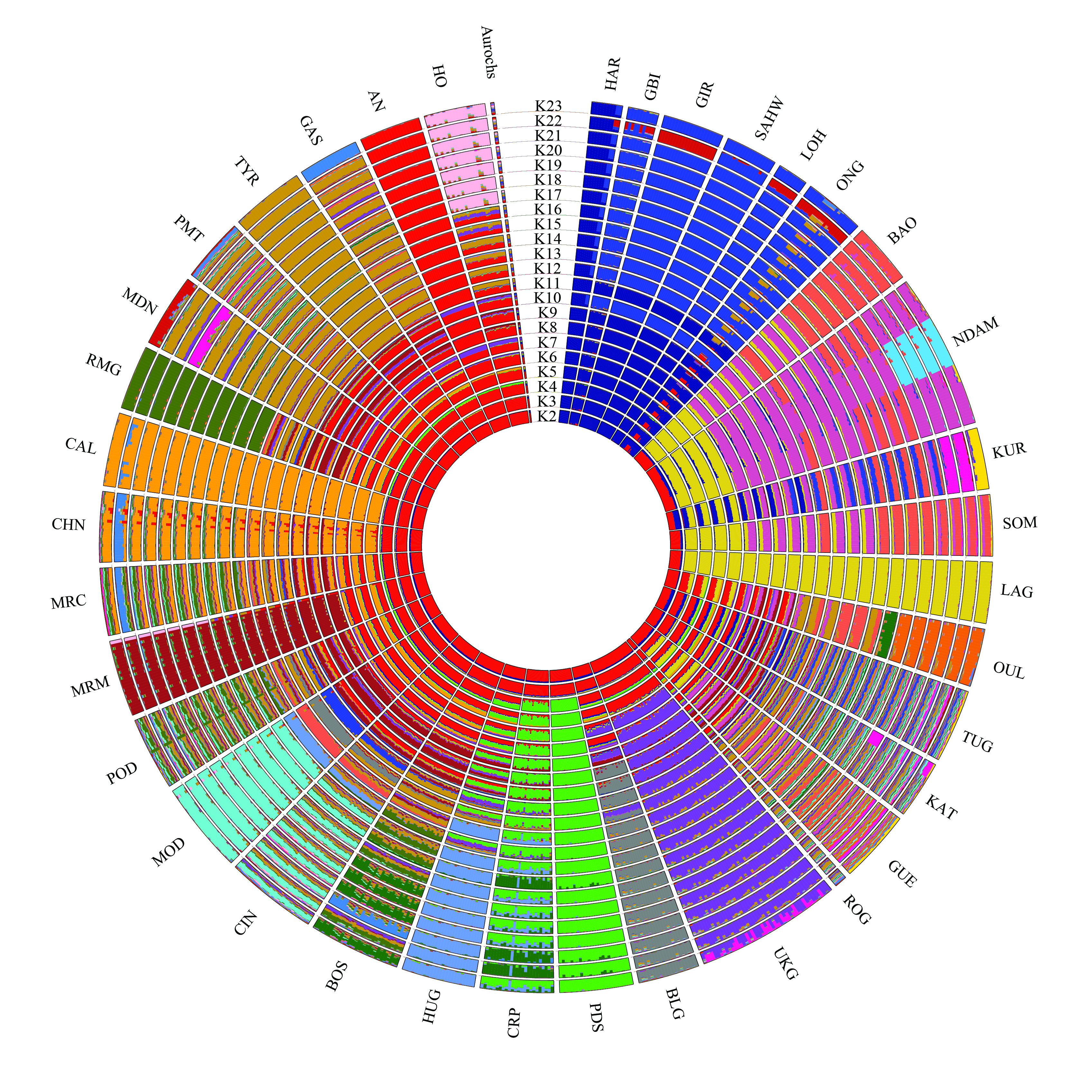

Supplement: Supplementary file 6 — Additional file 6: Figure S4. Admixture analysis plot in a circular fashion with all values of K (number of clusters) ranging from 2 to 23. [file 12711_2021_639_MOESM6_ESM.jpg]

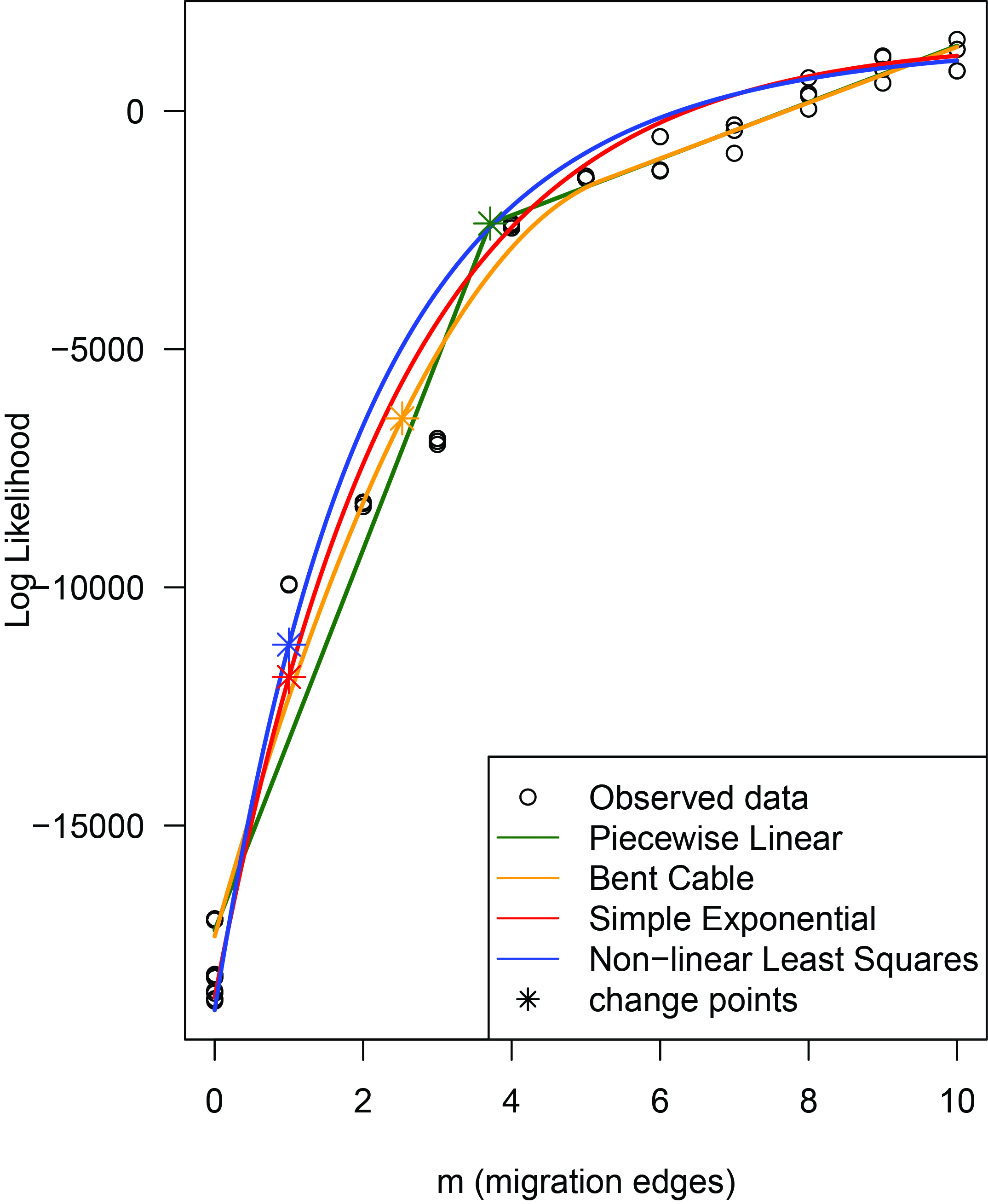

Supplement: Supplementary file 7 — Additional file 7: Figure S5. Increment in the log likelihood for the complete dataset for all tested migration events, calculated by using the optM function in the R package OptM. [file 12711_2021_639_MOESM7_ESM.jpg]

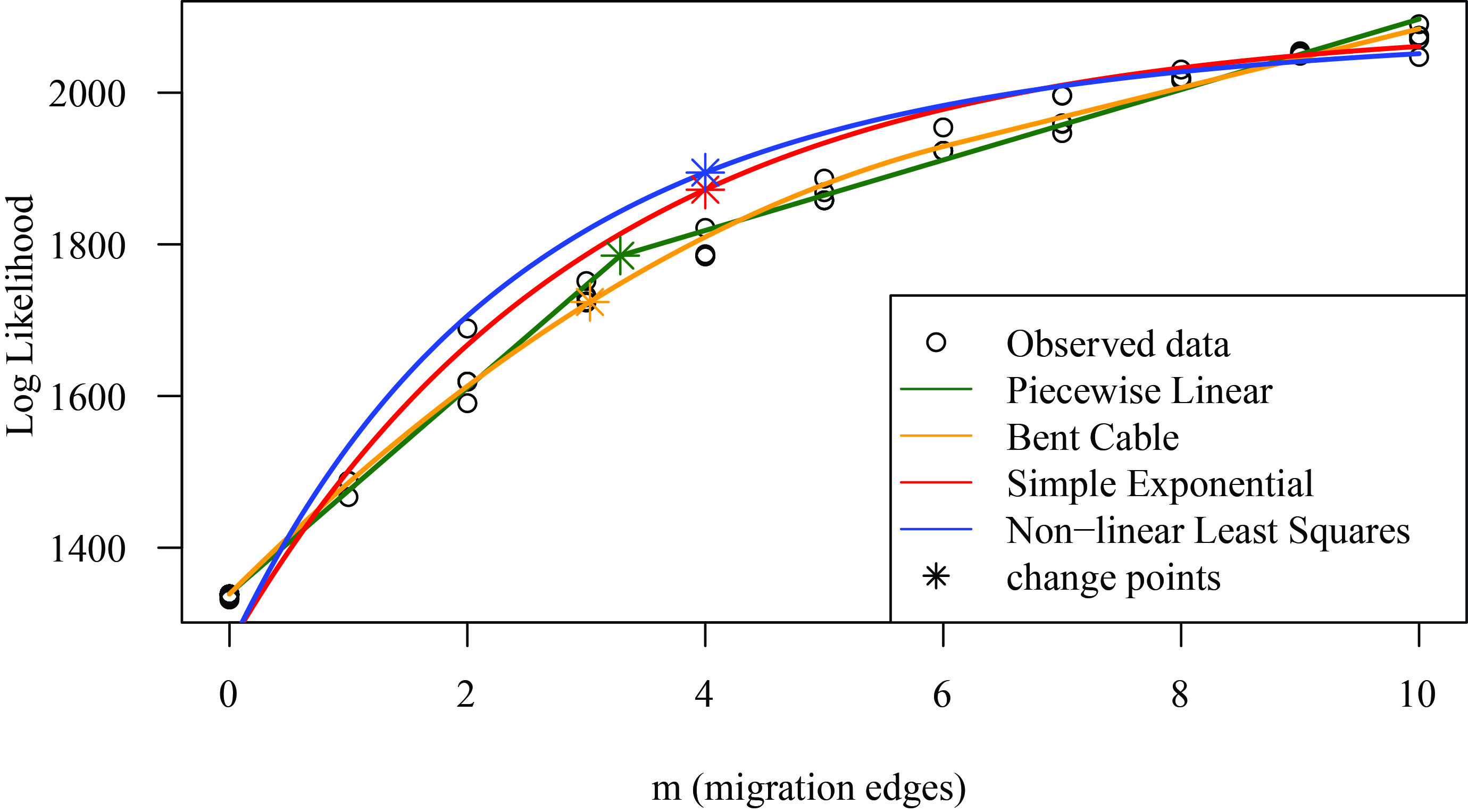

Supplement: Supplementary file 8 — Additional file 8: Figure S6. Increment in the log likelihood for the reduced (European taurine and the Guelmoise) dataset for all tested migration events, calculated by using the optM function in the R package OptM. [file 12711_2021_639_MOESM8_ESM.jpg]

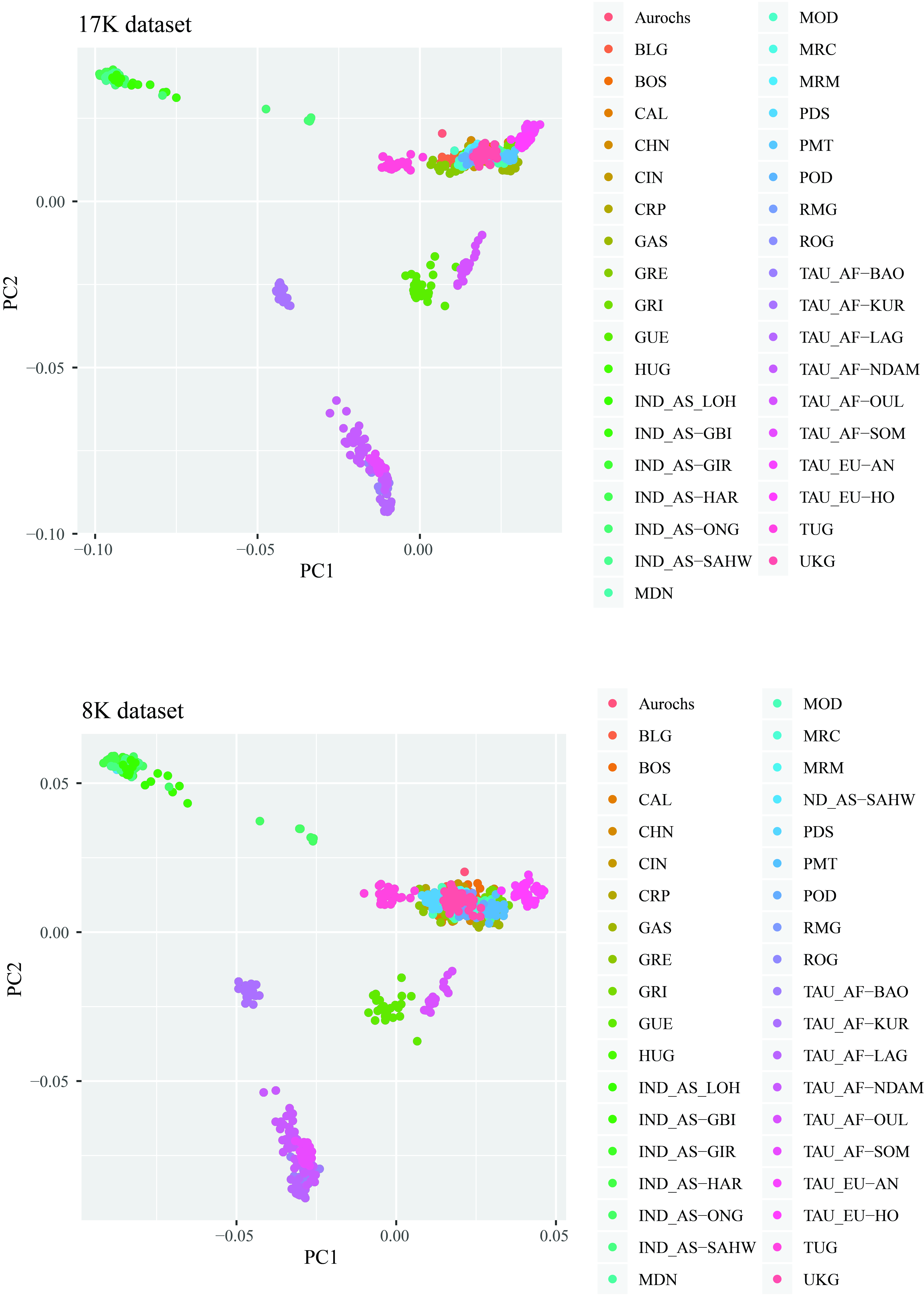

Supplement: Supplementary file 9 — Additional file 9: Figure S7. Principal component analysis (PCA) for the two datasets used (17 K and 8 K). [file 12711_2021_639_MOESM9_ESM.jpg]

a)

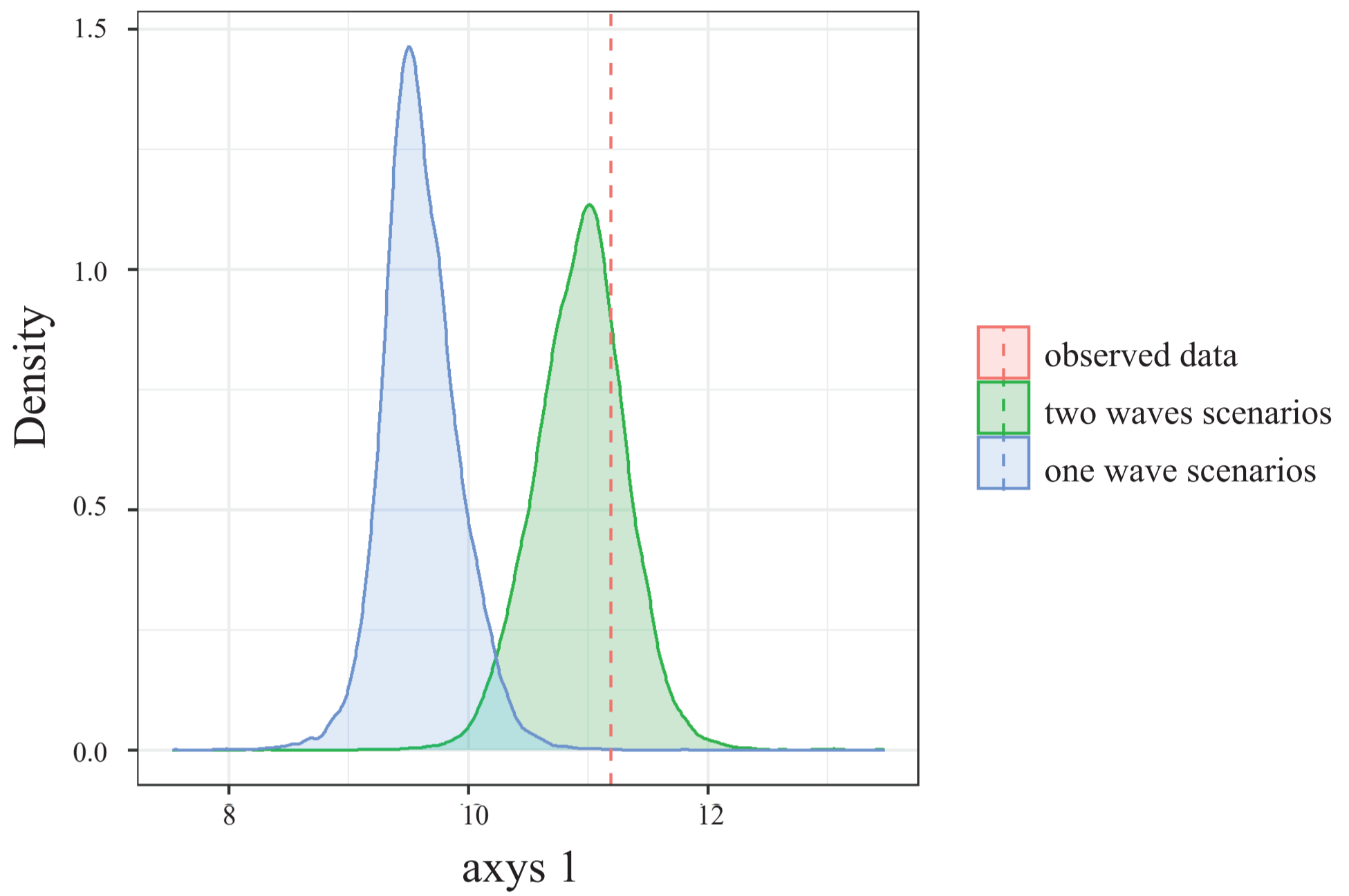

b)

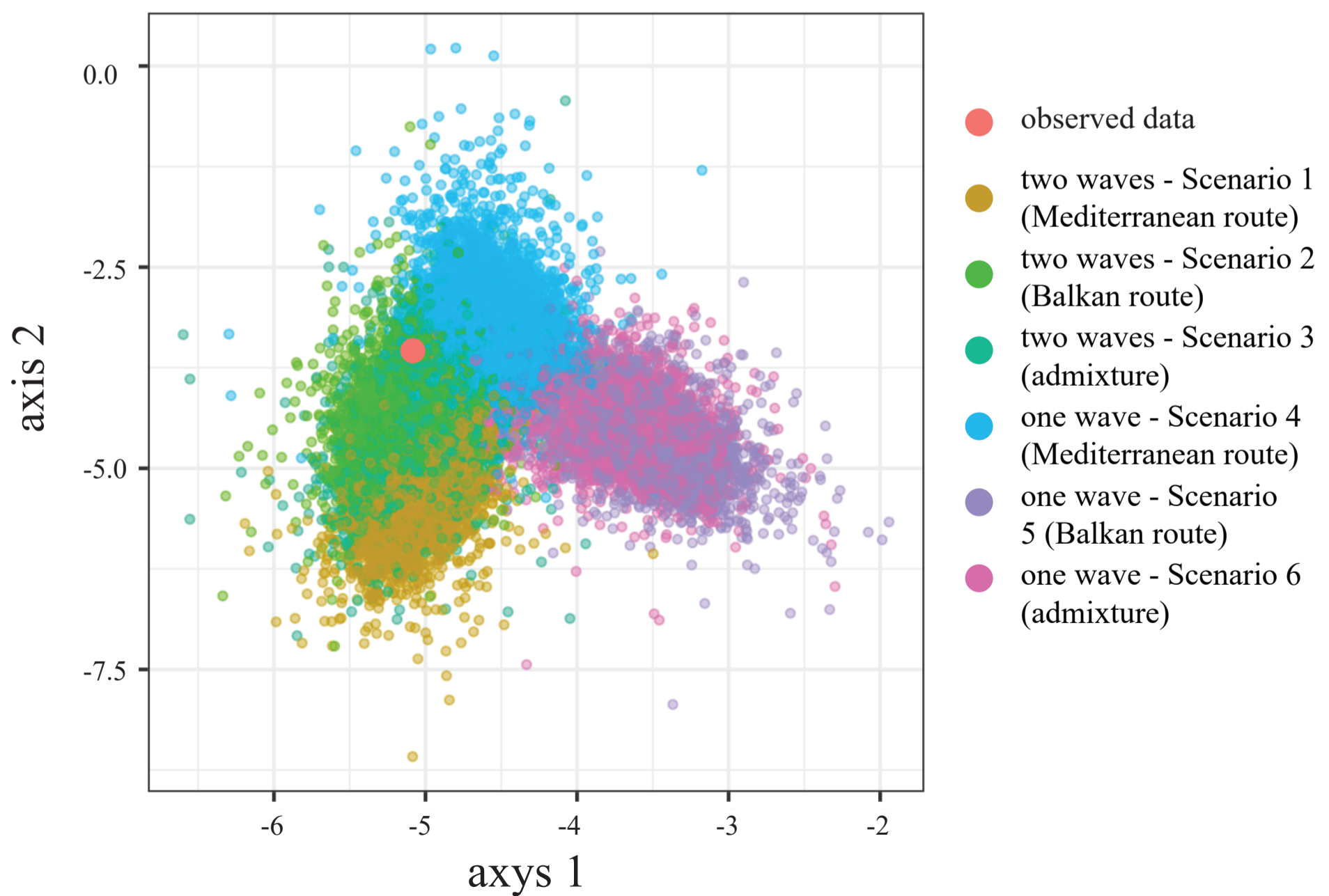

Supplement: Supplementary file 10 — Additional file 10: Figure S8. Projection on a single LDA axis in the model-grouping approach (a) and on the first two LDA axes in the six scenarios separately (b). [file 12711_2021_639_MOESM10_ESM.pdf]
