# Supplementary material for: On the origin and diversification of Podolian cattle breeds: testing scenarios of European colonization using genome-wide SNP data
Source: Genet Sel Evol. 2021 Jun 2;53:48. doi: 10.1186/s12711-021-00639-w (PMC8173809; doi:10.1186/s12711-021-00639-w)
Supplement: Supplementary file 3 — Additional file 3: Table S2. Set of priors used to model the scenarios in the ABC framework. [file 12711_2021_639_MOESM3_ESM.docx]

**Table S2**. Set of prior used to model the scenarios in the ABC framework.

| **Parameter** | **Distribution** | **Min.** | **Max.** |
| --- | --- | --- | --- |
| N1 | uniform | 10 | 50,000 |
| N2 | uniform | 10 | 50,000 |
| N3 | uniform | 10 | 50,000 |
| N4 | uniform | 10 | 50,000 |
| N5 | uniform | 10 | 50,000 |
| N6 | uniform | 10 | 50,000 |
| Na | uniform | 10 | 50,000 |
| N2b | uniform | 10 | 50,000 |
| N4b | uniform | 10 | 50,000 |
| N5b | uniform | 10 | 50,000 |
| t1 | uniform | 10 | 10,000 |
| t2 | uniform | 10 | 20,000 |
| t3 | uniform | 10 | 30,000 |
| ta | uniform | 10 | 20,000 |
| ta1 | uniform | 10 | 10,000 |
| tdi | uniform | 10 | 10,000 |
| tdt | uniform | 3,600 | 7,800 |
| tc | uniform | 10 | 10,000 |
| ra | uniform | 0.05 | 0.95 |
|  |  |  |  |
